# Comparative proteomic analysis reveals alterations in development and photosynthesis-related proteins in diploid and triploid rice

**DOI:** 10.1186/s12870-016-0891-4

**Published:** 2016-09-13

**Authors:** Shuzhen Wang, Wenyue Chen, Changdeng Yang, Jian Yao, Wenfei Xiao, Ya Xin, Jieren Qiu, Weimin Hu, Haigen Yao, Wu Ying, Yaping Fu, Jianxin Tong, Zhongzhong Chen, Songlin Ruan, Huasheng Ma

**Affiliations:** 1 Laboratory of Plant Molecular Biology & Proteomics, Institute of Biotechnology, Hangzhou Academy of Agricultural Sciences, Hangzhou, 310024 China; 2 State Key Laboratory of Rice Biology, China National Rice Research Institute, Hangzhou, 310006 China; 3 Jiaxing Academy of Agricultural Sciences, Jiaxing, 314016 China; 4 Department of Agronomy, College of Agriculture and Biotechnology, Zhejiang University, Hangzhou, 310012 China

**Keywords:** Rice, Polyploidy, Photosynthesis-related proteins, TMT, Morphology, Differential proteomics

## Abstract

**Background:**

Polyploidy has pivotal influences on rice (*Oryza sativa* L.) morphology and physiology, and is very important for understanding rice domestication and improving agricultural traits. Diploid (DP) and triploid (TP) rice shows differences in morphological parameters, such as plant height, leaf length, leaf width and the physiological index of chlorophyll content. However, the underlying mechanisms determining these morphological differences are remain to be defined. To better understand the proteomic changes between DP and TP, tandem mass tags (TMT) mass spectrometry (MS)/MS was used to detect the significant changes to protein expression between DP and TP.

**Results:**

Results indicated that both photosynthesis and metabolic pathways were highly significantly associated with proteomic alteration between DP and TP based on biological process and pathway enrichment analysis, and 13 higher abundance chloroplast proteins involving in these two pathways were identified in TP. Quantitative real-time PCR analysis demonstrated that 5 of the 13 chloroplast proteins ATPF, PSAA, PSAB, PSBB and RBL in TP were higher abundance compared with those in DP.

**Conclusions:**

This study integrates morphology, physiology and proteomic profiling alteration of DP and TP to address their underlying different molecular mechanisms. Our finding revealed that ATPF, PSAA, PSAB, PSBB and RBL can induce considerable expression changes in TP and may affect the development and growth of rice through photosynthesis and metabolic pathways.

**Electronic supplementary material:**

The online version of this article (doi:10.1186/s12870-016-0891-4) contains supplementary material, which is available to authorized users.

## Background

Polyploidy is a prevalent biological phenomenon in the chromosomal evolution of extant species and genera [1, 2], including the major crop plants such as rice, maize, wheat, soybean, and cotton. Most plant species have polyploid ancestries [3], and polyploidy may have played a critical role in flowering plant diversification [4]. Polyploid genotypes may lead to the differences in morphology, physiology and molecular characteristics, etc. Physiological traits, such as cell size, plant height (PH), growth rate, flowering time and fertility, can be altered by polyploidization [5]. Miller and coworkers’ research suggests that ploidy can affect flower size, stomatal size and seed weight [6]. Compared with the corresponding diploids (DPs), autopolyploids tend to have larger cells, resulting in the enlargement of some organs, such as leaves, flowers and seeds [7, 8]. Chao and coworker discover that polyploid *Arabidopsis* exhibit resistance to salinity and higher potassium uptake [9]. Some other changed traits, such as pest resistance, apomixes, drought tolerance, flowering time and organ size, can also contribute to the success of polyploids in agriculture [10, 11].

Besides offering evolutionary flexibility and phenotypic diversity for newly formed polyploids, polyploidy has considerable impacts on chromosomal rearrangement, nuclear enlargement and epigenetic changes, leading to the restructuring of the transcriptome, metabolome and proteome [12]. The epigenetic and developmental alterations allow polyploids to establish new species and promote their niches in local environments through restructuring genome and regulatory networks [13]. Polyploidy plays a key role in duplicating gene expression, and many of these expression alterations are organ-specific [14]. Blanc and Wolfe propose that the functional diversification of duplicated genes is a major characteristic of long-term polyploidy events in *Arabidopsis thaliana* [15]. Polyploidy also has important impacts on genome structure and gene expression [16, 17]. DNA methylation changes are observed in allopolyploids and their progenitors in many plants [18–21]. However, little is known about the complex nature of polyploidy [22].

Interestingly, large differences in morphology and physiology, including PH, leaf size and color, and chlorophyll content, are shown among rice with different ploidies, such as haploid (HP), DP and triploid (TP) rice. Besides, these differences are obviously amplified by the increase of ploidy level. The gene expression differences between HP and DP rice have been well documented [23], and the proteomic alterations during rice hull development are demonstrated by our recent research [24]. However, the proteomic changes between DP and TP in rice are poorly understood.

Thus, to test the impacts of polyploidy on rice development and chloroplast protein expression, we used tandem mass tags (TMT)-based proteomic methods to quantitatively screen the differentially expressed proteins among DP and TP. Meanwhile, chloroplast proteins were further analyzed to evaluate the influences of photosynthesis on DP and TP rice plants. In addition, quantitative real-time PCR (qRT-PCR) was used to verify the reliability of the chloroplast-related proteins with differential expressions. Through these approaches, our results may provide a global insight into the associated proteomic alterations in chloroplast and the impacts of ploidy on rice traits.

## Results

### Phenotypes of DP and TP

**Fig. 1:**
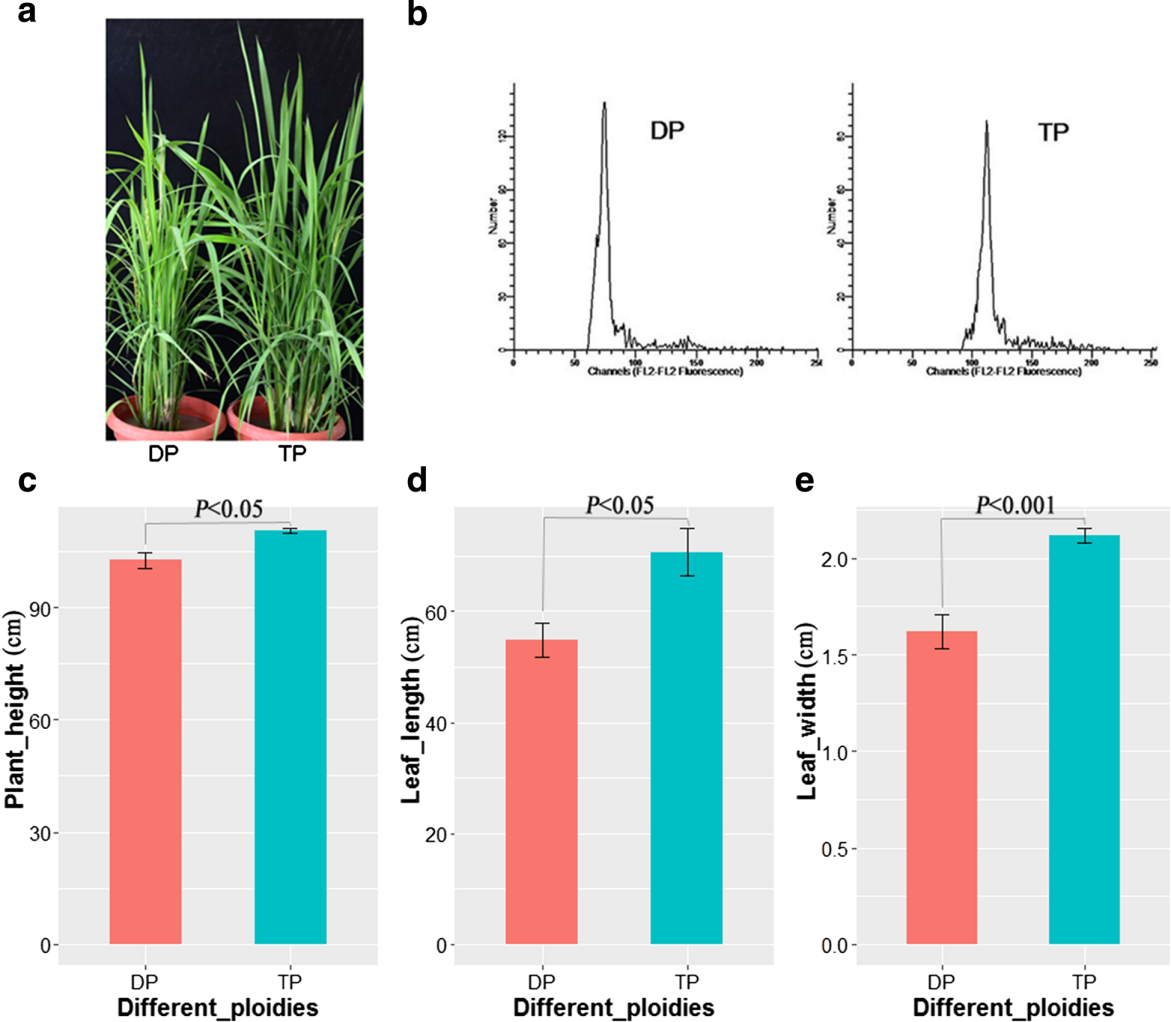
Phenotypes and growth indexes of diploid and triploid rice plants. **a** DP showed smaller plant and lighter leaf color compared to TP; **b** The flow cytometry of DP and TP rice plants; **c** The PH of DP and TP rice plants; **d** The LLs of DP and TP rice plants; **e** The LWs of DP and TP rice plants. (PH: plant height; LL: leaf length; LW: leaf width; DP: diploid; TP: triploid)

**Fig. 2:**
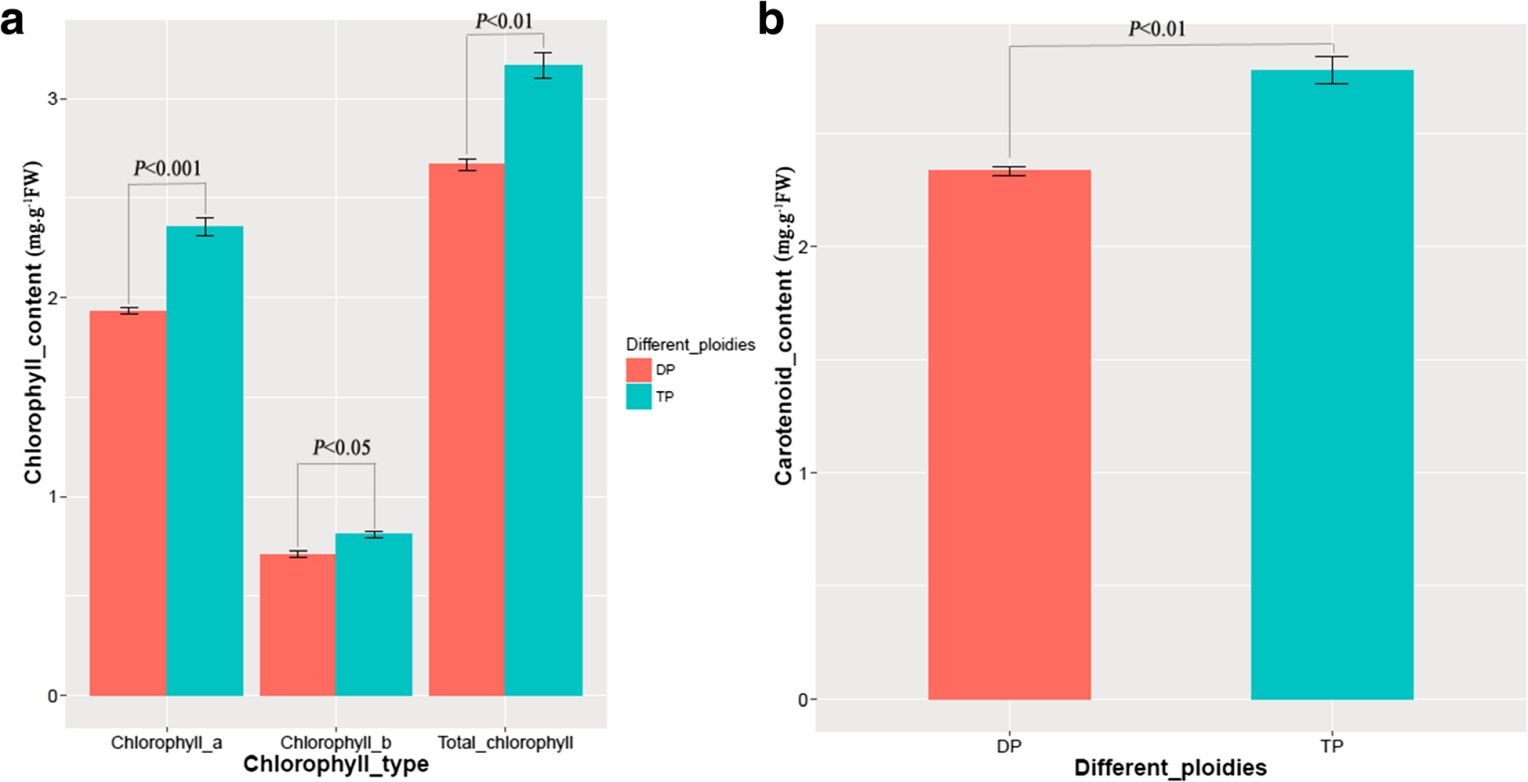
The chlorophyll and carotenoid contents of diploid and triploid rice plants. **a** The chlorophyll contents of DP and TP rice plants; **b** The carotenoid contents of DP and TP rice plants. DP: diploid; TP: triploid

To identify the phenotypes of rice plants between DP and TP, nuclear DNA ploidy analysis was firstly performed by flow cytometry to identify DP and TP (Fig. 1b). The increases of PH, LL and LW were positively correlated with ploidy levels (Fig. 1). The values of PH, LL and LW in TP were significantly larger than those in DP (Fig. 1c, d, e). Similarly, the contents of chlorophyll and carotenoid were higher in TP than in DP (Fig. 2).

### Comparative proteomic analysis of biological process in DP and TP

**Fig. 3:**
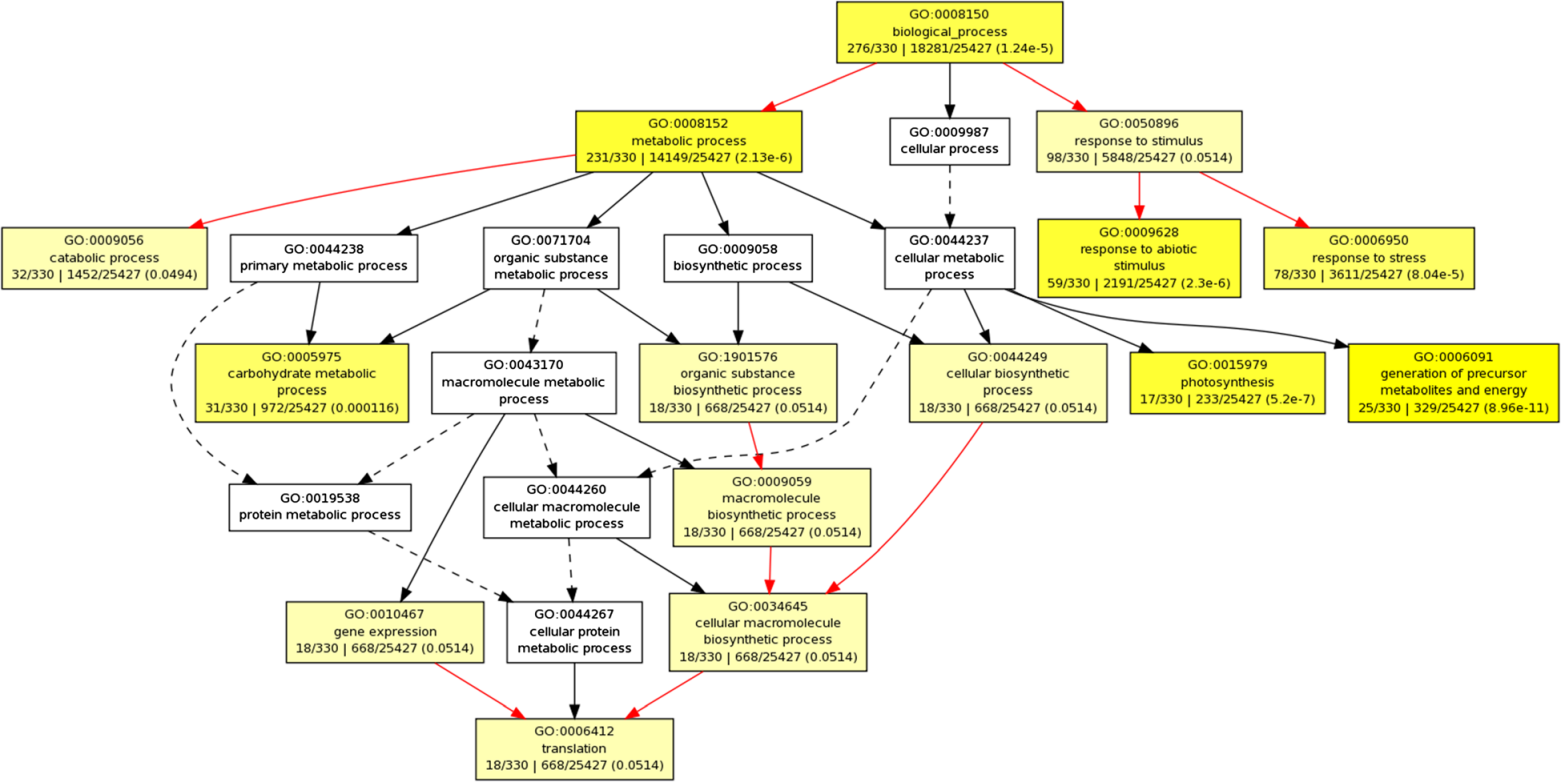
Biological process enrichment analysis based on the differentially expressed proteins of diploid and triploid rice plants (*p* < 0.05). DP: diploid; TP: triploid

Of the 1256 identified proteins, 365 differentially expressed proteins (fold change >1.5) showed the global false discovery rate <0.01 with ≥95 % confidence. Compared with their expressions in DP, 311 proteins were up-regulated and 54 were down-regulated in the TP. To uncover the different biological mechanisms between DP and TP, we annotated the differentially expressed proteins with GO terms and conducted a GO biological process. Multiple significant biological process were found to be involved in the differentially expressed proteins between DP and TP (Fig. 3), including generation of precursor metabolites and energy (GO:0006091, *p* = 8.96 × 10^−11^), photosynthesis (GO:0015979, *p* = 5.2 × 10^−7^), metabolic process (GO:0008152, *p* = 2.13 × 10^−6^), response to abiotic stimulus (GO:0009628, *p* = 2.3 × 10^−6^), response to stress (GO:0006950, *p* = 8.04 × 10^−5^), carbohydrate metabolic process (GO:0005975, *p* = 1.2 × 10^−4^), and catabolic process (GO:0009056, *p* = 0.0494).

### Pathway analysis

**Fig. 4:**
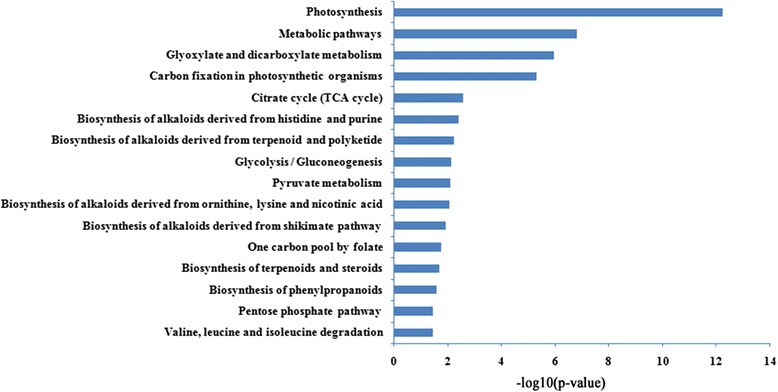
KEGG pathway enrichment analysis based on the differentially expressed proteins of diploid and triploid rice plants (*p* < 0.05). DP: diploid; TP: triploid

To identify potential protein targets, we performed pathway analysis on differentially expressed proteins using KEGG databases in rice plants with DP and TP (Fig. 4). Our results demonstrated that 16 significant pathways were enriched at the 5 % significant level. Among these significant pathways, photosynthesis, metabolic pathways, glyoxylate and dicarboxylate metabolism, and carbon fixation in photosynthetic organisms were highly significant (*p* < 0.001) associated with the differentially expressed proteins between DP and TP. Both photosynthesis and metabolic pathways were found to be related to alterations of protein expression in the development between DP and TP according to GO biological process and pathway enrichment analysis.

### Analysis of differentially expressed chloroplast proteins and qRT-PCR validation

Chloroplast plays a crucial role in conducting photosynthesis and regulating and regulating metabolic biological process. To demonstrate the roles of chloroplast in rice ploidy, we studied the protein expression alterations in the chloroplasts of DP and TP. Chloroplast proteins CYB6, ribulose bisphosphate carboxylase large chain (RBL), Apocytochrome f (CYF), 3 ATP synthase subunits ATPA, ATPB and ATPF, 4 photosystem II reaction center proteins PSBB, PSBC, PSBD and PSBH, as well as 3 photosystem I related proteins PSAA, PSAB and PSAC were shown to have differentially up regulated in TP compared with DP (Table 1). None of these chloroplast proteins was differentially down-regulated in TP. Among these chloroplast proteins, 12 and 13 proteins were involved in photosynthesis and metabolic pathways, respectively; while three, one and one proteins were associated with oxidative phosphorylation, glyoxylate and dicarboxylate metabolism, and carbon fixation in photosynthetic organisms, respectively.

**Table 1:** The pathways participated by the differentially expressed chloroplast proteins between diploid and triploid rice plants

Protein Name	Description	Pathways	FC^f^﻿
CYB6	Cytochrome b6	osa00195^a^;osa01100^b^	1.80
TPB	ATP synthase subunit beta, chloroplastic (EC 3.6.3.14) (ATP synthase F1 sector subunit beta) (F-ATPase subunit beta)	osa00190^c^;osa00195^a^; osa01100^b^	1.79
RBL	Ribulose bisphosphate carboxylase large chain (RuBisCO large subunit) (EC 4.1.1.39)	osa00630^d^;osa00710^e^; osa01100^b^	1.99
PSBH	Photosystem II reaction center protein H (PSII-H) (Photosystem II 10 kDa phosphoprotein)	osa00195^a^;osa01100^b^	2.84
CYF	Apocytochrome f	osa00195^a^;osa01100^b^	1.66
PSBD	Photosystem II D2 protein (PSII D2 protein) (EC 1.10.3.9) (Photosystem Q(A) protein)	osa00195^a^ osa01100^b^	2.15
PSBC	Photosystem II CP43 reaction center protein (PSII 43 kDa protein) (Protein CP-43) (Protein P6)	osa00195^a^;osa01100^b^	1.97
PSBB	Photosystem II CP47 chlorophyll apoprotein (PSII 47 kDa protein) (Protein CP-47)	osa00195^a^;osa01100^b^	1.71
PSAC	Photosystem I iron-sulfur center (EC 1.97.1.12) (9 kDa polypeptide) (PSI-C) (Photosystem I subunit VII) (PsaC)	osa00195^a^ osa01100^b^	2.60
PSAB	Photosystem I P700 chlorophyll a apoprotein A2 (EC 1.97.1.12) (PSI-B) (PsaB)	osa00195^a^;osa01100^b^	2.09
PSAA	Photosystem I P700 chlorophyll a apoprotein A1 (EC 1.97.1.12) (PSI-A) (PsaA)	osa00195^a^;osa01100^b^	1.64
ATPA	ATP synthase subunit alpha, chloroplastic (EC 3.6.3.14) (ATP synthase F1 sector subunit alpha) (F-ATPase subunit alpha)	osa00190^c^;osa00195^a^; osa01100^b^	1.79
ATPF	ATP synthase subunit b, chloroplastic (ATP synthase F(0) sector subunit b) (ATPase subunit I)	osa00190^c^;osa00195^a^; osa01100^b^	2.74

^a^Osa00195 represents photosynthesis; ^b^osa01100 represents metabolic pathways; ^c^osa00190 represents oxidative phosphorylation; ^d^osa00630 represents glyoxylate and dicarboxylate metabolism; ^e^osa00710 represents carbon fixation in photosynthetic organisms; ^f^FC fold change

**Fig. 5:**
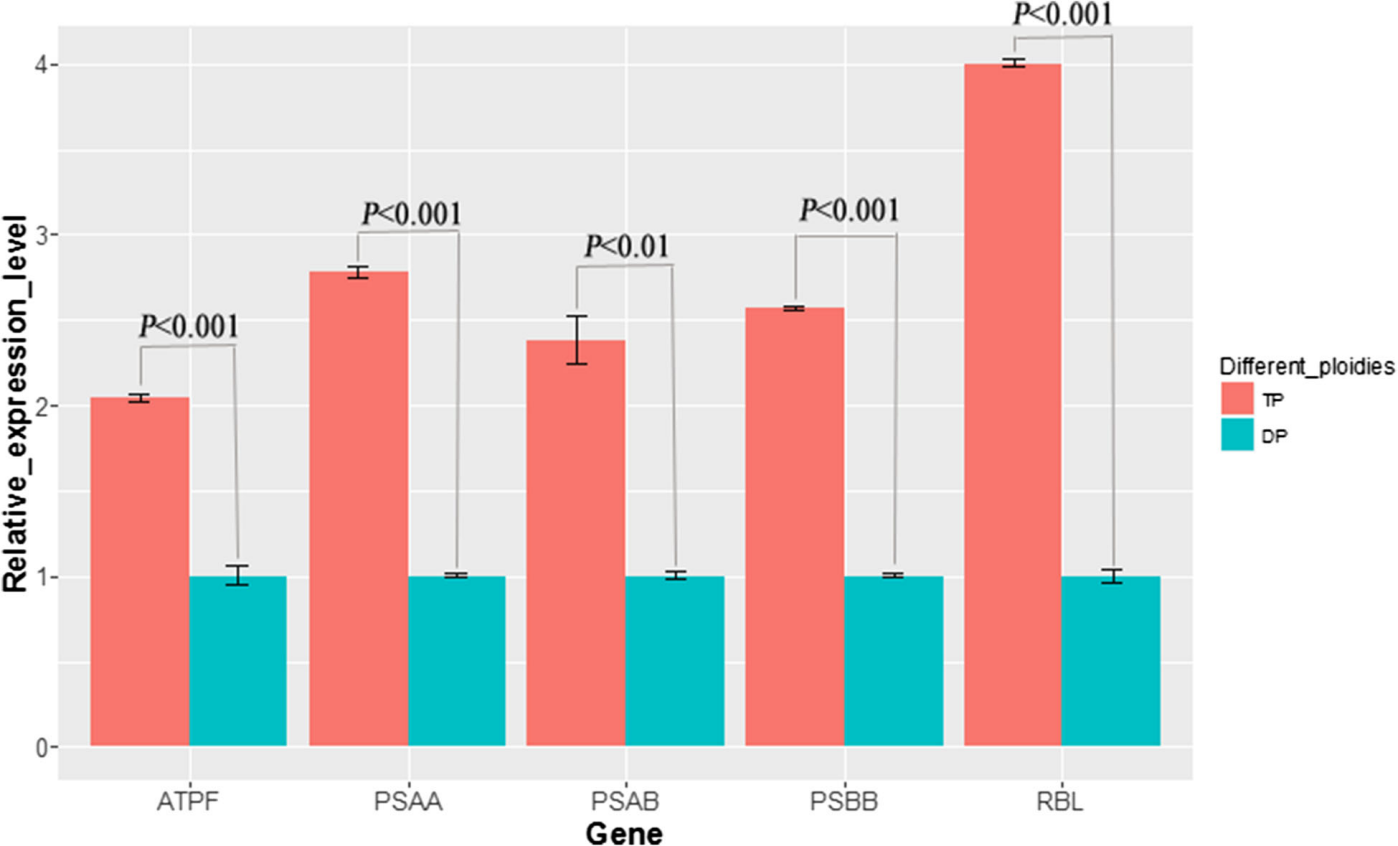
Independent validation of the differentially expressed chloroplast proteins by qRT-PCR between diploid and triploid rice plants (*p* < 0.05)

qRT-PCR was performed to validate the transcriptional levels for differentially expressed proteins among DP and TP (Fig. 5). qRT-PCR results showed that five of the 13 chloroplast genes were differentially transcripted between TP and DP, including *ATPF*, *PSAA*, *PSAB*, *PSBB* and *RBL* (Fig. 5). All five genes were associated with metabolic pathways, and *ATPF*, *PSAA*, *PSAB* and *PSBB* were related to photosynthesis.

## Discussion

Ploidy is a common feature and major factor of plant speciation. It drives the evolution of novel phenotypes and ecological tolerances [25]. Although the identification of candidate genes and developmental regulations in plant polyploids have been extensively pursued [16, 17, 23], a clear picture of proteins and pathways involved in regulatory and developmental differentiations has not been drawn for ploidy rice plants. In this study, our results suggests that proteomic alterations may account for the diversifications caused by ploidy in rice, and the significantly differentially expressed proteins and enriched pathways may help to unravel the complex underlying mechanisms in rice ploidy. Multiple pathways, especially photosynthesis and metabolic pathways, were found to be greatly significantly associated with proteomic alterations between DP and TP, indicating that photosynthesis and metabolic pathways account for major contribution to the proteomic differentiation.

Accumulating evidence demonstrates that chloroplasts participate in a variety of complex signaling pathways to regulate plant development, photosynthesis and metabolism with a exquisite way [26]. In Arabidopsis, chloroplast potassium efflux antiporters influence photosynthesis and growth of fully developed rosettes [27]. The critical role of chloroplasts is beyond dispute and has been reported in plant immunity recently [28, 29]. Recently, a chloroplast-localized protein LLB was found to affect the growth in rice [30]. Although it is well known that chloroplast affects the growth of rice, the underlying molecular mechanisms are not yet understood clearly. Consistent with the higher chlorophyll content in TP, 13 significantly differentially expressed chloroplast proteins were found to be up-regulated in TP, and 5 proteins were validated by qRT-PCR. Cytochrome *bc* complexes play key roles in respiration and photosynthesis [31]. The differential expression of CYB6 between the rice with different ploidies indicates that it may participate in energy transduction in respiratory membranes and photosynthesis. Among these validated proteins, ATPF belongs to a plant-specific protein family and is characterized by CRM domain, a recognized RNA binding domain [32]. RBL is a RubisCO large subunit, and the most abundant protein that serving as the major engine for carbon assimilation [33]. It underlines that TP may improve the efficiency of photosynthesis via coupling with the reaction of RBL. PSAA, PSAB, and PSBB are components of the photosystem II core complex which is a critical element of photosynthesis [34]. Currently, little is known about the functions of PSAA, PSAB and PSBB.

To the best of our knowledge, this is the first study to analyze the proteomic alterations of rice plants with different ploidies using TMT MS/MS technology. Our analysis suggested that TP tended to maintain their needs via more photosynthesis and metabolic activities than DP. Among the 13 candidate chloroplast proteins, 5 were validated. Through the combination of morphology, physiology and proteomic profiling, our results characterize the function of ATPF, PSAA, PSAB, PSBB and RBL in TP, and provide new insights for further understanding the molecular characteristics of rice ploidy.

## Conclusions

Both photosynthesis and metabolic pathways were highly significantly associated with proteomic alteration between DP and TP based on biological process and pathway enrichment analysis, and 13 up-regulated chloroplast proteins involving in these two pathways were identified in TP. This study integrates morphology, physiology and proteomic profiling alteration of DP and TP to address their underlying different molecular mechanisms. Our findings show that ATPF, PSAA, PSAB, PSBB and RBL can induce considerable expression changes in TP and may affect the development and growth of rice through photosynthesis and metabolic pathways.

## Methods

### Plant materials

To investigate the proteomic changes among rice plants with different ploidies, we sampled the leaves from eighty-day-old rice plants of DP and TP. Late uninucleate anthers collected from a rice strain (*O. sativa ssp japonica *cv H14) that was supplied by Jiaxing Academy of Agricultural Sciences (Jiaxing, China) using microscopic identification were cultured at 27 °C under the condition of 12 h light/dark photoperiod at light intensity of 2000 lx for one and half month on dedifferentiation medium containing N6, 1.0 mg/l 2,4-D, 3.0 mg/l NAA, 5.0 mg/l KT, 5 % (m/v) sucrose and 0.8 % (m/v) agar to produce calli with haploid cells. Then calli were transferred and cultured under the same conditions as above for two and half weeks on the differentiation medium with the same formula as dedifferentiation medium to regenerate the haploid seedlings. By spontaneous chromosome doubling, doubled haploid seedlings were produced, which could be grown into a doubled haploid (DP) plants. In the doubling process, a small amount of TP plants were obtained. All rice plants were cultivated in field during the period from June 15th, 2013 to October 10th, 2013. Three experimental replicates of each rice line were tested.

### Measurement of plant height (PH), leaf length (LL) and width (LW)

The heights of four replicates of 10 fifty-day-old plants were measured. Plant height was calculated by the distance from the basal part of stem to the tip of the highest leaf. The lengths and widths of 10 leaves from four replicates of fifty-day-old plants for each rice ploidy were measured. The standard errors (SE) of mean PH, LL or LW were calculated.

### Ploidy identification

A total of 20 mg of young leaf tissues were chopped with sharp scalpel in glass petri dish with 1 ml of Otto I buffer containing 0.1 M citric acid and 0.5 % Tween-20. The chopped materials were filtered with a 350 μm nylon filter and incubated for 10 min by stirring. Then, the nuclei in the filtrate were pelleted by centrifugation for 5 min at 150 × g, resuspended in 200 μl of Otto I buffer, and incubated at room temperature for 10 min. Subsequently, 500 μl of Otto II buffer containing 0.4 M sodium hydrogen phosphate, 5 μM propidium iodid (PI) and 50 μg/ml RNase was added to stain DNA. Samples were then analyzed within 1 h or stored at 4 °C for 24 h. All samples were analyzed with a Cell Lab Quanta^TM^ SC (Beckman Coulter Inc.) flow cytometer equipped with 488 nm diode laser for excitation. Data were collected by the corresponding software.

### Determination of chlorophyll contents

The contents of chlorophyll a and chlorophyll b were directly measured from the crude chlorophyll extracts of flag leaves. A total of 0.2 g leaf tissues were homogenized in ethanol at 4 °C as described by Porra et al. [35]. The homogenates were centrifuged and their fluorescence at 662, 645 and 470 nm was measured with a UV2550 Spectrometer.

### Protein preparation

One gram of fresh rice leaves were ground in liquid nitrogen and suspended in 5 ml acetone with 10 % (*w/v*) trichloroacetic acid and 0.07 % (*w/v*) β-mercaptoethanol at −20 °C for 1 h, followed by centrifugation for 15 min at 35,000 × *g*. The pellets were resuspended in acetone with 0.07 % (w/v) β-mercaptoethanol, incubated at −20 °C for 1 h, and then centrifuged for 15 min at 4 °C. This step was repeated for three times. Then, the pellets were lyophilized. The crude protein powders were solubilized in lysis buffer (8 M urea, 2 M thiourea, 4 % CHAPS, 0.5 % ampholine (pH 3–10), 50 mM DTT and 1 mM PMSF) for 1 h at room temperature, followed by centrifugation for 15 min at 15,000 × *g*. The supernatants were collected in 1.5 ml tubes, and 40 μl samples were used to detect protein concentrations by Bradford assay, with bovine serum albumin as the standard.

### Protein digestion and TMT labeling

A total of 100 μg of samples were digested during the FASP procedure [36], with little modification. Each sample was transferred to a 10 k filter (Pall Corporation) and centrifuged at 10,000 g at 20 °C for 20 min. A total of 200 μl UA buffer (8 M urea, 0.1 M Tris-HCl, pH 8.5) was added, and the samples were centrifuged at 14,000 g for 20 min again. Then, the sediments were mixed with 200 μl 100 mM IAA in UA buffer and incubated at room temperature in darkness for an additional 40 min. After that, IAA was removed by centrifugation at 14,000 g for 20 min, diluted with 200 μl UA buffer, and centrifuged twice. A total of 200 μl 0.5 M triethylammonium bicarbonate (TEAB) buffer (pH 8.5) was added and the samples were centrifuged at 14,000 g for 20 min. This step was repeated twice. Finally, the samples were digested at 37 °C for 20 h, and peptides were collected by centrifugation at 16,000 g. To increase the yield of peptides, the filter was washed twice with 500 μl 0.5 M TEAB buffer (pH 8.5). The peptide solutions were dried in vacuum concentrator.

The TMT labeling procedure was performed following the manufacturer’s instructions (Thermo Fisher Scientific). Briefly, for each 6-plex experiment, the reaction mixtures contained 25 μl TMT reagent and 75 μl (80 μg) tryptic digest in TEAB buffer to ensure reagent’s stability by limiting the organic (acetonitrile) content between 25 and 30 % (*v/v*). The peptides from DP and TP samples were labeled with reagents for three biological replicates. After labeling, the reaction mixtures were incubated at room temperature for 1.5 h, and then 8 μl of 5 % hydroxylamine solution was added to quench the labeling reaction. Then the TMT-modified digest from 6-plex experiment was combined into one sample and dried in vacuum.

### Peptide fractionation with strong cation exchange (SCX) chromatography

Sample fractionation was performed by SCX chromatography as previously described [37]. Briefly, the sample was resuspended in SCX buffer (7 mM KH_2_PO_4_, pH 2.6, 30 % ACN) and separated through 2.0 × 50 mm polySULFOETHYL A HPLC column (5 μm, 200 Å, PolyLC). Separation was performed by applying a gradient SCX buffer B (7 mM KH_2_PO_4_, 350 mM KCl, pH 2.6, 30 % ACN) from 0 to 50 % in 30 min at a flow rate of 0.1 ml/min, followed by 50 to 100 % SCX buffer A gradients and then buffer B in 10 min using an Agilent 1100 quaternary pump outfitted with degasser and photodiode array detector (PDA) (Thermo Scientific). Samples were collected in 5 min increments, and dried under vacuum. Fractions were then redissolved with 1 % FA and combined into a total of seven samples based on their intensities from SCX chromatographic UV trace. These samples were then desalted by C18 SPE and dried under vacuum.

### MS/MS analysis

RP-HPLC separation was performed on a nanoflow HPLC (Proxeon Biosystems, now Thermo Fisher Scientific) equipped with self-packed tip column (75 μm × 150 mm; C18, 3.0 μm) using a 120 min gradient at a flow rate of 300 nl/min. Q-Exactive mass spectrometer (Thermo Fisher Scientific) was used and equipped with nanoelectrospray ion source (Proxeon Biosystems, now Thermo Fisher Scientific). Data were acquired in the data-dependent “top10” mode in which the ten precursor ions with most abundance were selected with high resolution (70 000 at m/z 200) from the full scan (300–1800 m/z) for HCD fragmentation. Precursor ions with singly charged or unassigned charge information were excluded. The resolution for MS/MS spectra was set to 17 500 at m/z 200, target value was 2E5 (AGC control enabled) and isolation window was set to 2.0 m/z, with a lock mass option enabled for the 445.120025 ion [38]. The normalized collision energy was 29 %.

### Protein identification and the relative quantitation criteria

All MS/MS spectra were searched using the MaxQuant software [39]. The TMT tags on lysine residues, peptide N termini (229.162932 Da) and the carbamidomethylation of cysteine residues (57.02146 Da) were set as static modifications, while the oxidation of methionine residues (+15.99492 Da) was set as variable modification. The missing of two cleavage sites was allowed. The tolerances of peptides and fragment ions were set at 10 ppm and 20 ppm, respectively. The false discovery rate on peptides and proteins was fixed at no more than 0.01. Reporter ion quantitation was based on the extraction of the TMT reporter ion signals of each peptide by MaxQuant software. Proteins were then quantified by summing reporter ion counts across all peptide matches, and then normalized by assuming equal protein loadings across all samples. We used the following criteria to identify the differentially expressed proteins among DP and TP: (1) These proteins must have been examined in all 3 MS preparations; (2) They must have been verified with the confidence greater than 95 %; (3) The fold changes of their expressions should >1.5 or <2/3, and the significant differences (*p* < 0.05) in *t*-test should be reached.

### Biological process, pathway statistical analyses

Rice gene annotations were acquired from the Rice Annotation Project Database (RAP-DB) [40], the Michigan State University (MSU) Rice Genome Annotation [41] and UniProt [42]. Chloroplast proteins were identified from uniprot (www.uniprot.org). Gene Ontology (GO) [43] and GOEAST [44] were used for biological process analysis between DP and TP. The differentially expressed proteins between DP and TP were analyzed using KEGG pathway [45] to identify the molecular pathways that may have differential activities involved in DP and TP. Two-tailed Student’s t-tests were conducted to determine whether there are differences between DP and TP, including plant height, leaf length, width, chlorophyll content, carotenoids level and mRNA abundance levels. All statistical analyses were performed in R environment, using several CRAN packages (http://cran.r-project.org/).

### Validation of protein expression by qRT-PCR

Frozen leaf tissue was homogenized in liquid nitrogen using a mortar and pestle. Total RNA was extracted using Trizol according to the supplier’s recommendation (Invitrogen, Karlsruhe, Germany). Residual DNA was removed with an RNase-free DNase (Fermentas, EU). One microgram total RNA was reverse-transcribed using 0.5 μg of Oligo (dT) 20 and 200 units of ReverTra Ace (TOYOBO, Japan) following the supplier’s recommendation. qRT-PCR assays were performed to validate the expression changes of chloroplast proteins among DP and TP. Relative gene expression levels were quantified based on cycle threshold (Ct) values and normalized to the reference proteins Tubulin and glyceraldehydes 3-phosphate dehydrogenase. The experiment for each sample was repeated for three technique replicates and the qRT-PCR results were calculated by means of three replications. Gene expression levels were calculated by 2-^△△Ct^ method. Six pairs of primers were designed for gene-specific transcript amplification (Additional file 1).

## Additional file

Additional file 1: Six pairs of primers were designed for gene-specific transcript amplification. (DOC 25 kb)

## Abbreviations

ATPA: ATP synthase subunit alpha
ATPB: ATP synthase subunit beta
ATPF: ATP synthase subunit b
CYB6: Cytochrome b6
CYF: Apocytochrome f
DP: Diploid
HP: Haploid
LL: Leaf length
LW: Width
MS: Mass spectrometry
PH: Plant height
PSAA: Photosystem I P700 chlorophyll a apoprotein A1
PSAB: Photosystem I P700 chlorophyll a apoprotein A2
PSAC: Photosystem I iron-sulfur center
PSBB: Photosystem II CP47 chlorophyll apoprotein
PSBD: Photosystem II CP43 reaction center protein
PSBE: Cytochrome b559 subunit alpha
PSBH: Photosystem II reaction center protein H
RBL: Ribulose bisphosphate carboxylase large chain
SCX: Strong cation exchange
TMT: Tandem mass tags
TP: Triploid
