# Supplementary material for: Comparative proteomic analysis reveals alterations in development and photosynthesis-related proteins in diploid and triploid rice
Source: BMC Plant Biol. 2016 Sep 13;16(1):199. doi: 10.1186/s12870-016-0891-4 (PMC5020550; doi:10.1186/s12870-016-0891-4)
Supplement: Additional file 1: — Six pairs of primers were designed for gene-specific transcript amplification. (DOC 25 kb) [file 12870_2016_891_MOESM1_ESM.doc]

The following primers were designed for gene-specific transcript amplification:

ATPF-F: 5’-AATGCCACTTCTATTAGTTTGG-3’,

ATPF-R: 5’-GCTCCTTGTACGGCTTGTT-3’;

PSAA-F: 5’- GGAGGTGGCGAGTTAGTAG-3’,

PSAA-R: 5’-AAGATTTGCTTTATCGGGTA-3’;

PSAB-F: 5’-ATCATATCTCATTTAAGTTGG-3’,

PSAB-R: 5’- ATCTCCAGGTCCTATTGTTA-3’;

PSBB-F: 5’- TAGTGTCCGTCCACCTCA-3’,

PSBB-R: 5’- GCTGACCCATACCACATAG-3’;

RBL-F: 5’- TTACAAAGGCCGATGCTA-3’,

RBL-R: 5’- CGTTACCCACAATGGAAGT-3’;

Tubulin-F: 5’- TACCGTGCCCTTACTGTTCC-3’,

Tubulin-R: 5’- CGGTGGAATGTCACAGACAC -3’.
